# Adropin reduces blood glucose levels in mice by limiting hepatic glucose production

**DOI:** 10.14814/phy2.14043

**Published:** 2019-04-19

**Authors:** Dharendra Thapa, Bingxian Xie, Janet R. Manning, Manling Zhang, Michael W. Stoner, Brydie R. Huckestein, Lia R. Edmunds, Xueyang Zhang, Nikolaos L. Dedousis, Robert M. O'Doherty, Michael J. Jurczak, Iain Scott

**Affiliations:** ^1^ Division of Cardiology Department of Medicine University of Pittsburgh Pittsburgh Pennsylvania; ^2^ Division of Endocrinology and Metabolism Department of Medicine University of Pittsburgh Pittsburgh Pennsylvania; ^3^ Department of Medicine Vascular Medicine Institute University of Pittsburgh Pittsburgh Pennsylvania; ^4^ Department of Medicine Center for Metabolism and Mitochondrial Medicine University of Pittsburgh Pittsburgh Pennsylvania

**Keywords:** Adropin, hepatic glucose production, hyperinsulinemic‐euglycemic clamp, insulin sensitivity, liver

## Abstract

Adropin is a liver‐ and brain‐secreted peptide hormone with striking effects on fuel metabolism regulation in a number of tissues. Previous studies demonstrated that adropin secretion is decreased in obese mice subjected to a long‐term high‐fat diet (HFD), and that whole‐body loss of adropin expression resulted in systemic insulin resistance. Treatment of obese mice with adropin improves glucose tolerance, which has been linked to increased glucose oxidation and inhibition of fatty acid utilization in isolated skeletal muscle homogenates. In this study, we used in vivo physiological measurements to determine how treatment of obese mice with adropin affects whole‐body glucose metabolism. Treatment with adropin reduced fasting blood glucose and, as shown previously, increased glucose tolerance in HFD mice during standard glucose tolerance tests. Under hyperinsulinemic‐euglycemic clamp conditions, adropin treatment led to a nonsignificant increase in whole‐body insulin sensitivity, and a significant reduction in whole‐body glucose uptake. Finally, we show that adropin treatment suppressed hepatic glucose production and improved hepatic insulin sensitivity. This correlated with reduced expression of fatty acid import proteins and gluconeogenic regulatory enzymes in the liver, suggesting that adropin treatment may impact the pathways that drive vital aspects of hepatic glucose metabolism.

## Introduction

Adropin is a liver‐ and brain‐derived peptide that elicits powerful metabolic effects on a number of diverse tissue types. Initially identified as a major regulator of liver metabolism (Kumar et al. 2008), adropin has also been shown to control metabolic processes in the brain and cardiovascular system. Adropin production is suppressed by obesity in mice and humans (Kumar et al. 2008; Butler et al. 2012), and its deletion in mice leads to increased adiposity, insulin resistance, and hepatosteatosis (Ganesh‐Kumar et al. 2012; Chen et al. 2017). In the vasculature, adropin treatment improved endothelial function (Lovren et al. 2010; Sato et al. 2018), and increased arterial stiffness is associated with reduced adropin levels in older adults and obese children (Fujie et al. 2015; Zhang et al. 2017). Finally, in the brain, adropin regulates drinking and physical activity (Wong et al. 2014; Stein et al. 2016).

More recent work has demonstrated that adropin has significant effects on energy substrate metabolism, and a particular focus has been on its function in different muscle cell types. Using a combination of in vivo and in vitro analysis, Butler and colleagues have shown that adropin regulates fuel substrate preference in skeletal muscle (Gao et al. 2014, 2015). Of note, these studies showed that adropin treatment could improve glucose homeostasis, and restore glucose utilization in the skeletal muscle of obese, insulin‐resistant mice by partially downregulating fatty acid oxidation (Gao et al. 2015). Exogenous adropin treatment impacted the expression of several mitochondrial fuel metabolism enzymes in vivo (Gao et al. 2014, 2015), and these effects have been further supported by similar findings using cardiac cells in vitro (Thapa et al. 2018).

In this study, we further examined the role of adropin in glucose homeostasis in vivo. Treatment of obese mice improved fasting glucose levels and glucose tolerance in obese mice; however, this did not correlate with improved skeletal muscle glucose uptake under hyperinsulinemic conditions. Instead, we found that adropin moderately suppressed basal hepatic glucose production, and improved liver insulin sensitivity during hyperinsulinemic‐euglycemic clamp studies. We conclude that these findings point to an underappreciated role for adropin in the regulation of hepatic glucose production.

## Materials and methods

### Animal care and use

Animal experiments were approved by the University of Pittsburgh Institutional Animal Care and Use Committee. Male C57BL/6J low‐fat diet (LFD) control (stock #380056) and diet‐induced obese mice (stock #380050) were obtained from The Jackson Laboratory aged 22–23 weeks, maintained on low‐fat diet (LFD; 70% carbohydrate, 20% protein, 10% fat; Research Diets D12450B) or high‐fat diet (HFD; 20% carbohydrate, 20% protein, 60% fat; Research Diets D12492) for 3–4 weeks while acclimating to their new environment, and used at 26 weeks of age (20 weeks total on HFD where appropriate).

### Animal surgery and in vivo procedures

**Figure 1:**
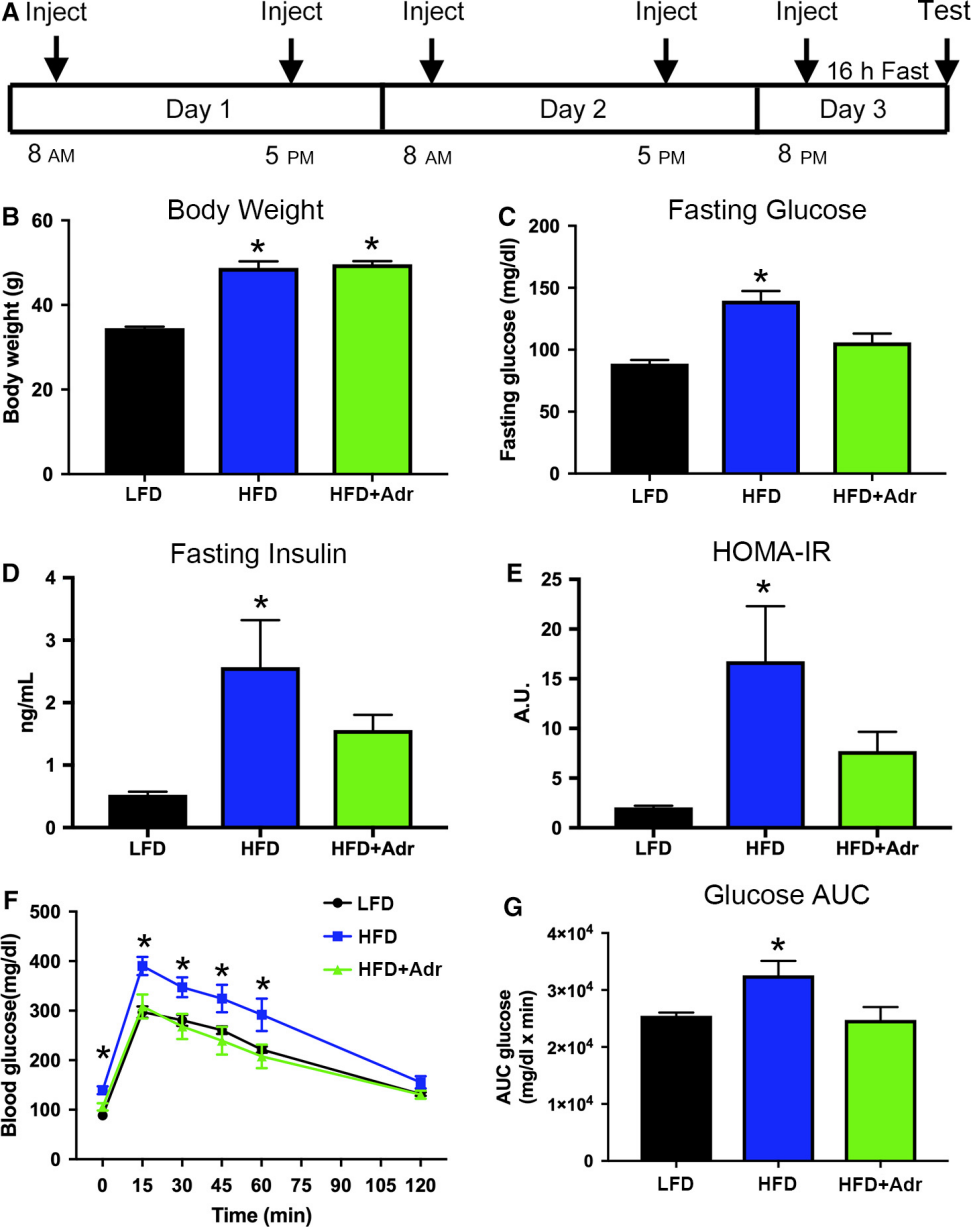
Adropin reduces fasting blood glucose and improves glucose tolerance in obese mice. (A) Injection schedule for adropin treatment prior to in vivo metabolism experiments. (B) Body weight was significantly increased in vehicle‐ and adropin (Adr)‐treated high‐fat diet (HFD) mice relative to low‐fat diet (LFD) controls. (C–E) Plasma glucose and insulin levels, along with HOMA‐IR, were significantly increased in vehicle‐treated HFD mice following an overnight fast. This increase was significantly attenuated in obese mice treated with adropin for 3 days. (F, G) Glucose tolerance was significantly improved in adropin‐treated HFD mice relative to vehicle‐treated obese controls. *N* = 6; *P < 0.05 versus LFD fed.

Mice received i.p. injections (b.i.d.) of vehicle (PBS) or adropin (450 nmol/kg) for 2 days, followed by a single i.p. injection on the morning of the third day prior to in vivo procedures (Fig. 1A).

Hyperinsulinemic‐euglycemic clamps were performed as previously described, with minor modifications (Costa et al. 2016). Mice recovered for 1 week prior to clamp experiments following surgical implantation of an indwelling catheter in the right jugular vein. Mice were fasted 6 h prior to infusion with [3‐^3^H] glucose at a rate of 0.05 *μ*Ci/min for 120 min to determine basal glucose turnover. A primed infusion of insulin and [3‐^3^H] glucose was administered at 10.7 mU/kg/min and 0.24 *μ*Ci/min, respectively, for 4 min, after which rates were reduced to 4.5 mU/kg/min insulin and 0.1 *μ*Ci/min [3‐^3^H] glucose. Blood was collected by tail massage for plasma measurements at set time points, and a variable infusion of 20% dextrose was given to maintain euglycemia. A bolus injection of [1‐^14^C] 2‐deoxyglucose (10 *μ*Ci) was given at 55 min during steady state to determine tissue‐specific rates of glucose transport. Glucose turnover was calculated as the ratio of the [3‐^3^H] glucose infusion rate to the specific activity of plasma glucose at the end of the basal infusion and during the last 40 min of the hyperinsulinemic infusion. Endogenous or hepatic glucose output represents the difference between the glucose infusion rate and the rate of glucose appearance. The plasma decay curve and tissue levels of the [1‐^14^C] 2‐deoxyglucose tracer were used to calculate tissue‐specific glucose transport rates. After the final blood sample, mice were euthanized with an intravenous injection of 150 mg/kg pentobarbital sodium.

Glucose tolerance tests (GTTs) were performed after an overnight fast as previously described (Jurczak et al. 2011) with minor modifications. After collecting a basal plasma sample (*t* = 0), mice were injected intraperitoneally with a 2 g/kg glucose bolus and blood glucose was measured by tail bleed at set time points using a Bayer Contour Next EZ handheld glucometer (*t* = 15, 30, 45, 60, and 120 min). HOMA‐IR was calculated using the standard equation (fasting insulin x fasting glucose/22.5) (Kumar et al. 2008; Gao et al. 2015).

### Western blotting

Liver tissue was rapidly harvested following sacrifice, washed in ice‐cold PBS, and flash‐frozen in liquid nitrogen. Tissues were homogenized using CHAPS lysis buffer using a bead mill, then incubated on ice for 60 min. Whole cell lysates were recovered by centrifugation at 4°C/13,000***g*** for 5 min. Protein lysates were prepared in LDS sample buffer, separated using Bolt SDS/PAGE 4–12% or 12% Bis‐Tris gels, and transferred to nitrocellulose membranes (Life Technologies). Protein expression was analyzed using the following primary antibodies: rabbit GAPDH (2118S) from Cell Signaling Technologies; rabbit PDK4 (PA5‐13776) from ThermoFisher; goat PGC‐1*α* (ab106814) from Abcam; rabbit CD36 (18836‐1‐AP), rabbit CPT1a (15184‐1‐AP), rabbit PEPCK1 (16754‐1‐AP), and rabbit G‐6‐Pase (22169‐1‐AP) from ProteinTech. Protein loading was confirmed using GAPDH as a loading control. Images were obtained and quantified using the LiCor Odyssey system.

### Peptide synthesis

The adropin (34–76) peptide was synthesized by the solid‐phase on Liberty Microwave Synthesizer using a FMOC synthesis protocol as previously reported (Thapa et al. 2019). The final product was re‐purified by HPLC and confirmed by mass spectrometry.

### Statistics

Means ± SEM were calculated for all data sets. Data were analyzed using two‐tailed student's *t*‐tests or one‐way ANOVA (with Dunnett's post hoc tests) as appropriate. *P *<* *0.05 was considered significant.

## Results

### Adropin reduces fasting blood glucose and improves glucose tolerance in obese mice

Previous studies have shown that acute adropin treatment improves glucose homeostasis in diet‐induced obese mice fed a HFD for 18–20 weeks (Kumar et al. 2008; Gao et al. 2015). We first sought to replicate these studies under our experimental conditions to ensure technical fidelity. LFD and HFD mice were given serial i.p. injections of vehicle or adropin over 3 days, followed by a 16‐h fast (Fig. 1A). Both the HFD mouse groups showed significantly increased body weight compared to LFD‐fed controls after 20 weeks (Fig. 1B). Fasting blood glucose and insulin (along with HOMA‐IR) were significantly elevated in vehicle‐treated HFD mice, which was reversed by adropin treatment (Fig. 1C–E). Mice were then given a bolus i.p. injection of glucose, and their plasma glucose levels measured over the next 120 min. Whole‐body glucose clearance was significantly decreased in vehicle‐treated HFD mice relative to LFD‐fed controls, while adropin‐treated HFD mice showed no change relative to LFD‐fed controls (Fig. 1F,G). Overall, these data support and extend upon previous studies showing that exogenous adropin treatment improves glucose tolerance in obese mice.

### Adropin treatment has negligible effects on whole‐body insulin sensitivity, while reducing whole‐body glucose uptake, in obese mice

**Figure 2:**
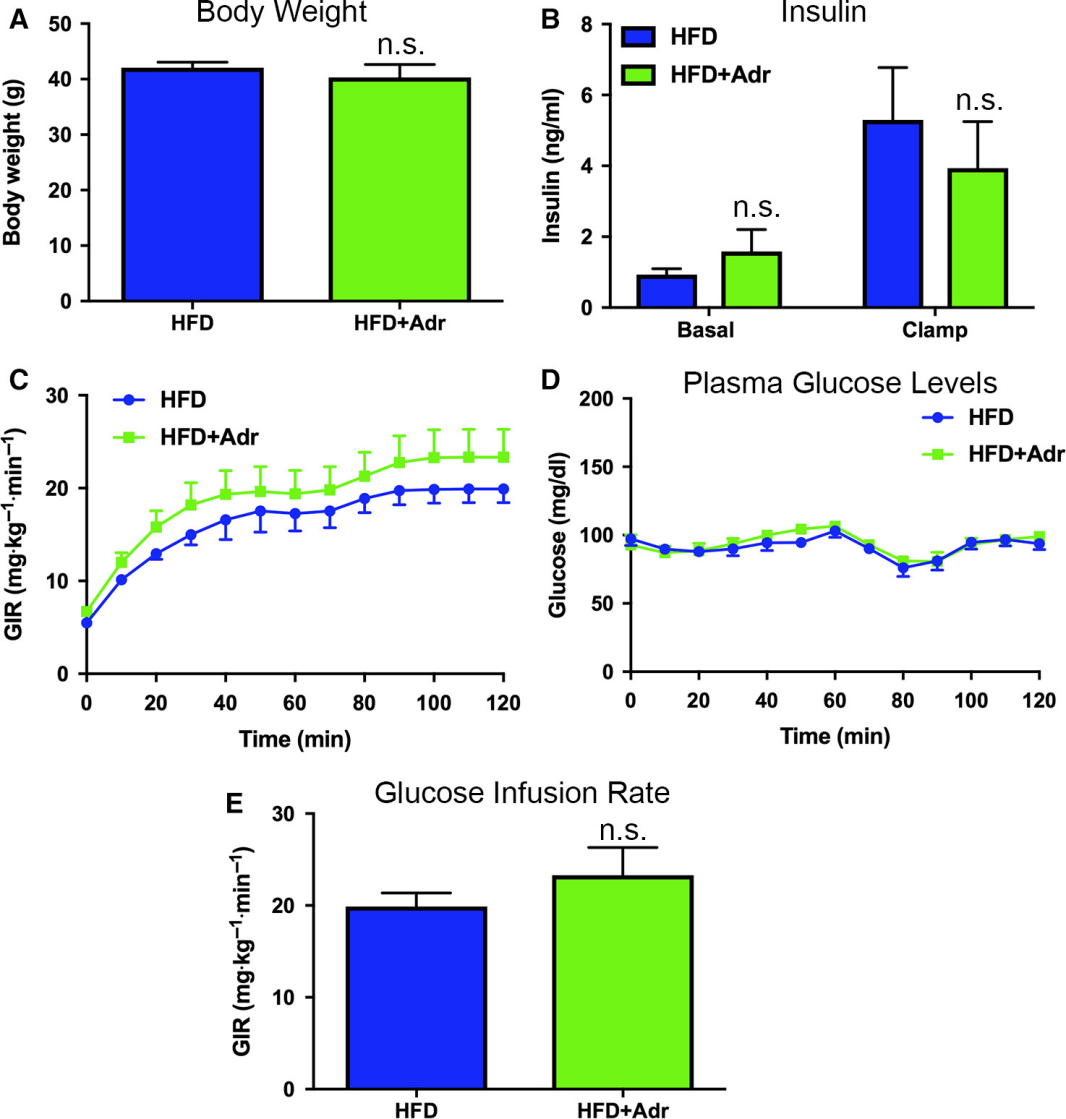
Adropin has minimal beneficial effects on whole‐body insulin sensitivity in obese mice. (A) Body weights were matched between vehicle‐ and adropin (Adr)‐treated high‐fat diet (HFD) mice groups. (B) There was no significant difference in plasma insulin levels under basal or hyperinsulinemic‐euglycemic clamp conditions. (C, D, E) Treatment with adropin led to a small, but nonsignificant improvement in whole‐body insulin sensitivity as shown by an increased glucose infusion rate required to maintain and match euglycemia between the groups over the course of the clamp. *N* = 6–8.

Previous studies demonstrated improved glucose tolerance in adropin‐treated obese mice, but could not account for tissue‐specific contributions to the observed improvements in whole‐body glucose homeostasis in vivo (Kumar et al. 2008; Gao et al. 2015). To better understand the action of adropin on glucose metabolism in obesity, we next examined insulin sensitivity, as well as whole‐body and tissue‐specific glucose metabolism in vehicle‐ and adropin‐treated mice using hyperinsulinemic‐euglycemic clamps. Body weights in both the HFD mouse groups were matched (Fig. 2A), and adropin treatment did not significantly affect insulin levels under basal or clamp conditions (Fig. 2B). Glucose infusion rates required to maintain euglycemia (Fig. 2C) in adropin‐treated HFD mice were 10% higher than in vehicle‐treated HFD controls during the clamp; however, this trend did not reach statistical significance (Fig. 2D,E). This suggests that adropin has only a moderate positive effect on whole‐body insulin sensitivity under hyperinsulinemic conditions.

**Figure 3:**
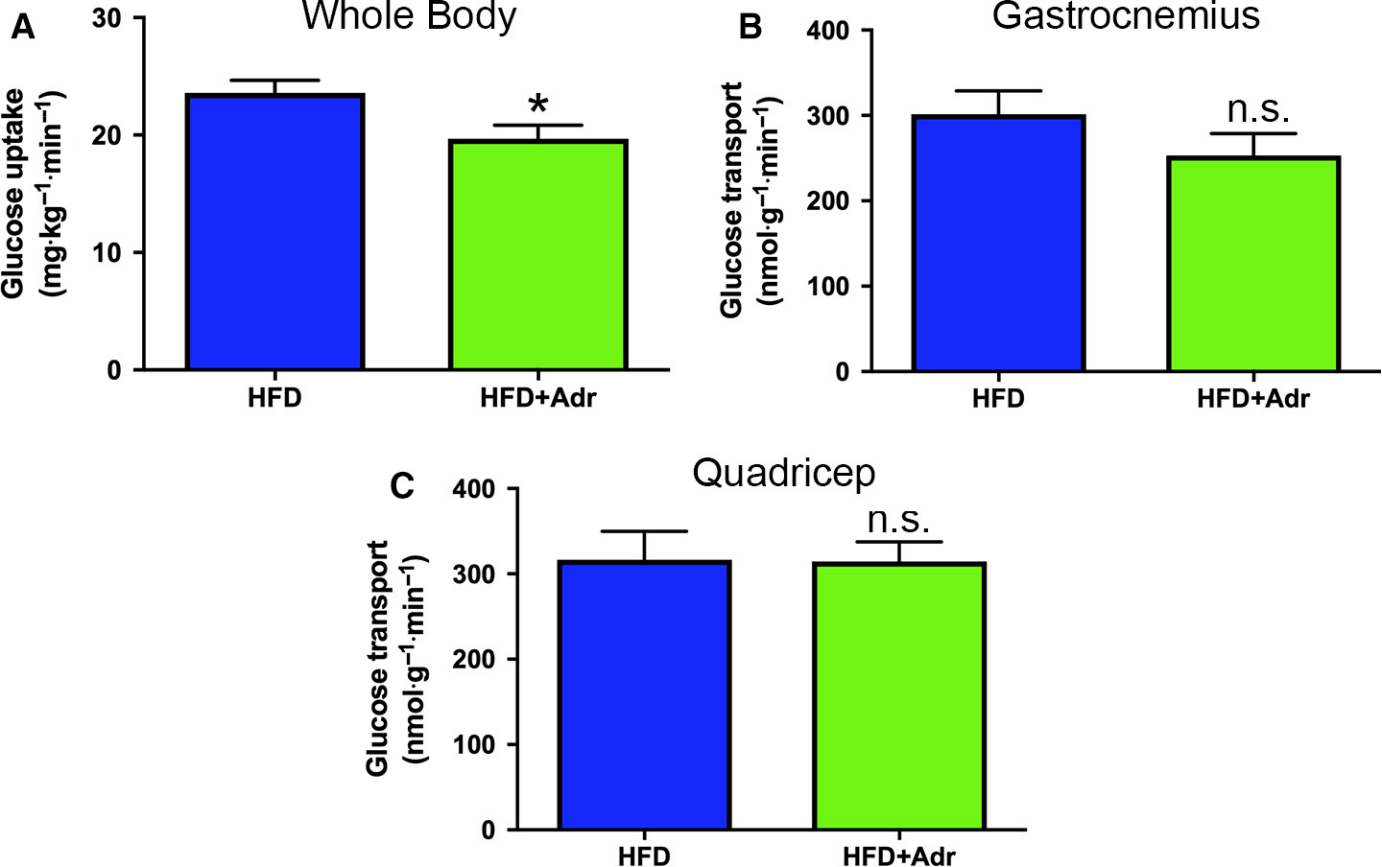
Adropin reduces whole‐body insulin‐stimulated glucose uptake in obese mice. (A) Whole‐body glucose uptake was significantly decreased in adropin (Adr)‐treated obese mice relative to high‐fat diet (HFD) controls under hyperinsulinemic conditions. (B) There was a small, but nonsignificant decrease in glucose uptake in the gastrocnemius of adropin‐treated HFD mice. (C) Furthermore, there was no change in quadricep glucose transport between the HFD mouse groups. *N* = 6–8; *P < 0.05 versus vehicle‐treated HFD.

Analysis of glucose turnover showed that adropin treatment significantly reduced whole‐body glucose uptake (Fig. 3A). This correlated to a nonsignificant ~15% decrease (*P *=* *0.23) in gastrocnemius glucose uptake (Fig. 3B). In contrast, uptake rates in the quadricep and heart remained unchanged (Fig. 3C, and data not shown). These data suggest that any moderate improvements in insulin sensitivity in adropin‐treated obese mice are not due to increased muscle glucose utilization under hyperinsulinemic conditions.

### Adropin lowers fasting hepatic glucose production and improves hepatic insulin sensitivity during hyperinsulinemic conditions

**Figure 4:**
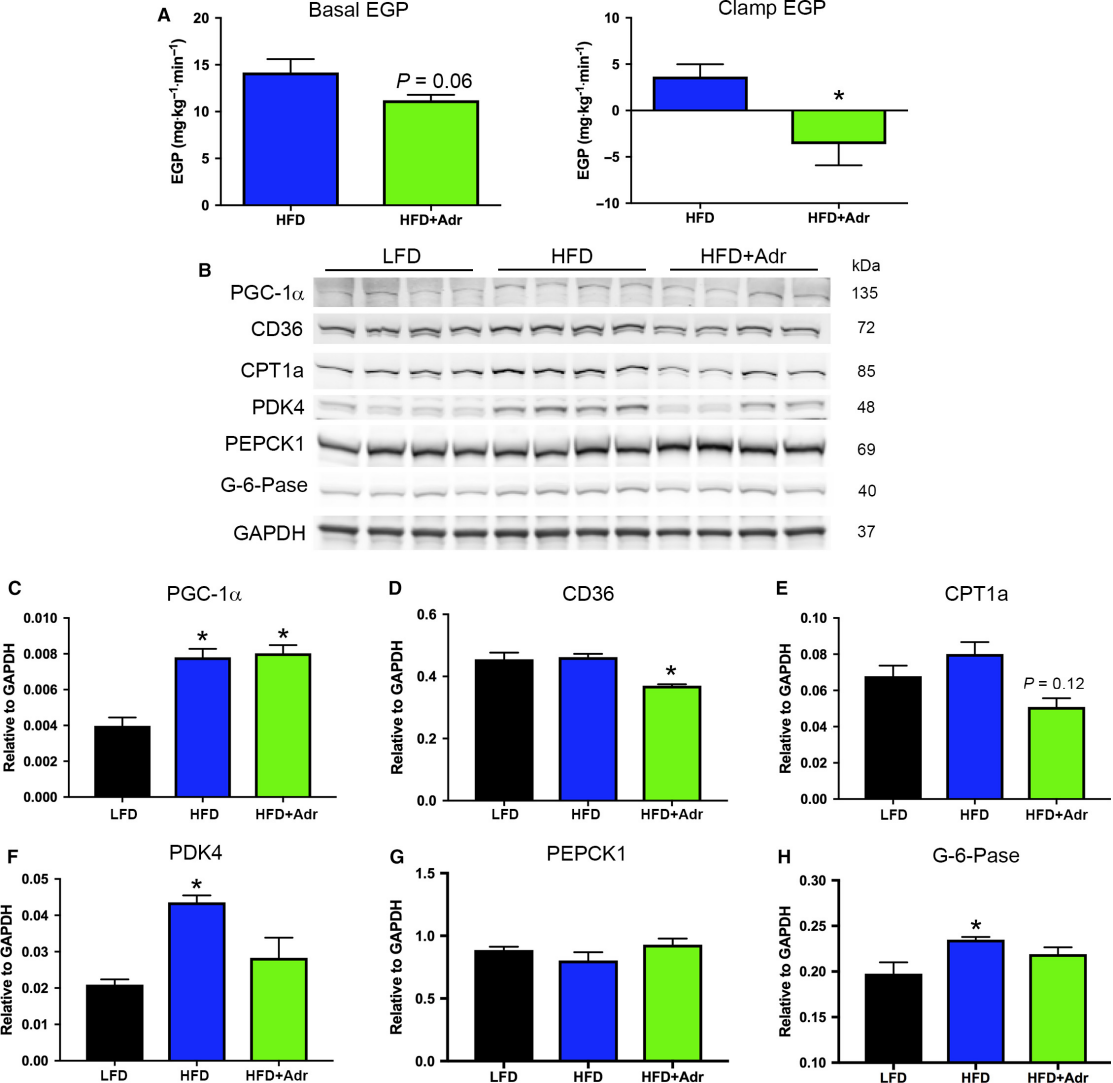
Adropin improves hepatic insulin sensitivity in obese mice during hyperinsulinemia. (A) There was a decrease in hepatic glucose production under both basal and hyperinsulinemic conditions in adropin (Adr) mice. (B–H). Adropin treatment suppressed expression of hepatic fatty acid uptake (CD36 and CPT1a), PDH‐inhibitory (PDK4), and gluconeogenic (G‐6‐Pase) proteins in high‐fat diet (HFD) diet mice. *N* = 4–8; *P < 0.05 versus vehicle‐treated HFD.

Finally, we examined other pathways that may promote improved glucose homeostasis in obese mice treated with adropin. Under basal fasting conditions, we found that there was a trend (*P *=* *0.06) toward reduced endogenous (hepatic) glucose production in adropin‐treated HFD mice relative to their vehicle‐treated controls (Fig. 4A). This trend was maintained under clamp conditions, and there was a significant reduction in hepatic glucose production in adropin‐treated mice relative to control animals (Fig. 4A). These data suggest that adropin reduces basal rates of hepatic glucose production and improves hepatic insulin sensitivity during hyperinsulinemia.

Adropin treatment of obese mice has been shown to limit fatty acid oxidation in skeletal muscle homogenates by reducing the expression of fatty acid uptake proteins, leading to a concurrent increase in glucose utilization (Gao et al. 2015). As fatty acid oxidation contributes to the energy required for hepatic glucose production (Rui 2014), we examined whether adropin treatment impacted mediators of fatty acid uptake in the liver. As expected, in both the HFD mouse groups there was a significant elevation of PGC‐1*α* expression relative to LFD mice (Fig. 4B,C). Despite this, there was a significant decrease in the expression of the cell membrane fatty acid uptake protein CD36, and a trend toward a decrease (*P *=* *0.12) in the expression of the mitochondrial fatty acid translocator CPT1a, in adropin‐treated HFD mice relative to vehicle‐treated obese controls (Fig. 4B,D,E). Additionally, while there was a significant upregulation of the pyruvate dehydrogenase inhibitory kinase PDK4 in HFD mice relative to LFD controls, this effect was lost following adropin treatment (Fig. 4B,F). Finally, while there was no change in the expression of the gluconeogenic enzyme PEPCK1, we found that there was a significant increase in G‐6‐Pase expression in HFD mice which was reversed by adropin treatment (Fig. 4B,G,H). Combined, these data point to a decrease in fatty acid utilization and reduced gluconeogenic activity as a potential mechanism for reduced hepatic glucose production in adropin‐treated obese mice.

## Discussion

In this report, we demonstrate that treatment of obese mice with adropin leads to improved glucose homeostasis. We show that adropin does not significantly impact whole‐body insulin sensitivity, and that the change in plasma glucose levels does not result from increased uptake of glucose in skeletal muscle. Instead, we show that adropin suppresses basal and insulin‐stimulated hepatic glucose production in obese mice, which may be linked to decreased fatty acid uptake utilization and gluconeogenesis in the liver. Combined, these data suggest that adropin has important effects on liver glucose metabolism, and may point to additional therapeutic avenues for this peptide in the control of metabolic dysfunction in obesity.

Using a combination of indirect in vivo calorimetry and in vitro biochemical assays, Butler and colleagues elegantly demonstrated that adropin treatment could restore insulin signaling and glucose oxidation in the skeletal muscle of obese mice (Gao et al. 2015). While our current results showed that adropin could modestly increase whole‐body insulin sensitivity in vivo, these changes were not significant (Fig. 2). Furthermore, we show that under hyperinsulinemic conditions, glucose uptake into white muscle was reduced by ~15%, correlating with a significant decrease in whole‐body glucose uptake (Fig. 3). Further work is required to understand if these limitations in skeletal muscle glucose uptake extend to defects in glucose oxidation in our experimental system in vivo.

Whole‐body adropin knockout (AdrKO) mice display increased adiposity, insulin resistance, and hepatosteatosis (Ganesh‐Kumar et al. 2012). Furthermore, under hyperinsulinemic‐euglycemic clamp conditions, it was shown that AdrKO mice have a greatly reduced capacity for insulin‐mediated suppression of hepatic glucose production in chow fed mice relative to wild‐type controls (Ganesh‐Kumar et al. 2012). In complementary studies, we show that adropin treatment of obese mice significantly increases their capacity to suppress endogenous glucose production in the liver (Fig. 4A). These combined findings may suggest that adropin has a key, and so far underappreciated, role in the regulation of hepatic glucose production that merits further investigation. This role is consistent with observed changes in plasma adropin levels during fasting and re‐feeding and subsequent changes in fuel utilization (Ganesh‐Kumar et al. 2012; Gao et al. 2015). During fasting, adropin levels are low, favoring peripheral fatty acid utilization to support hepatic glucose production, and glucose sparing to support the central nervous system. Conversely, during re‐feeding adropin levels increase favoring glucose utilization. As a first step, we show that the molecular machinery involved in fatty acid uptake in the liver is downregulated in adropin‐treated obese mice (Fig. 4B–F), which may point to effects on the bioenergetic pathways that drive hepatic gluconeogenesis (Rui 2014). There was also a decrease in the expression of the terminal gluconeogenic enzyme G‐6‐Pase in adropin‐treated HFD (Fig. 4B,H), which may point to reduced hepatic gluconeogenesis, however further studies will be required to understand the mechanism of this inhibition. Future studies using mass spectrometry‐based isotope tracers will allow us to determine whether the reduced glucose output is from inhibited gluconeogenesis alone, or involves other glucose producing pathways such as glycogenolysis.

In summary, these studies demonstrate that adropin treatment has the capacity to regulate hepatic glucose production in obese mice, and provides further premise for the continued investigation of this peptide in the therapeutic context of diabetes and metabolic dysfunction.

## Conflict of Interest

None declared.
